# Hand pathology in type 1 diabetes mellitus: a case-control study

**DOI:** 10.1007/s42000-025-00732-5

**Published:** 2025-11-10

**Authors:** Carolina Sager-La Ganga, Fernando Sebastian-Valles, Celia Gonzalez-Gonzalez, Mónica Marazuela, Miguel Antonio Sampedro-Nuñez, Jose Alfonso Arranz-Martín

**Affiliations:** Department of Endocrinology and Nutrition, Hospital Universitario de La Princesa Instituto de Investigación Sanitaria de La Princesa, Universidad Autónoma de Madrid, Madrid, 28006 Spain

**Keywords:** Diabetic hand, Type 1 diabetes mellitus, Glycemic control, Musculoskeletal complications, Case-control study

## Abstract

**Purpose:**

This study aims to investigate the factors associated with diabetic hand (DH) in patients with type 1 diabetes mellitus (T1D) using a matched case-control design.

**Methods:**

A retrospective case-control study was conducted at a tertiary care hospital in Spain. Among a cohort of 968 T1D patients, 45 cases of DH were identified. Cases were matched with 90 controls using propensity score matching based on age, sex, diabetes duration, and presence of retinopathy. Clinical data, comorbidities, and glycemic control (HbA1c and continuous glucose monitoring metrics) were collected. Multivariable conditional logistic regression was used to identify factors associated with DH.

**Results:**

DH prevalence was 4.6%. Significant associations were found for HbA1c (OR 1.96; 95% CI 1.16–3.28; *p* = 0.011) and age (OR 1.06; 95% CI 1.01–1.12; *p* = 0.012). Cardiovascular disease was more common in DH cases (20.2% vs. 2.2%; *p* = 0.005), but it was not independently associated after adjustment. No significant differences were observed for retinopathy, nephropathy, or other microvascular complications.

**Conclusions:**

Chronic poor glycemic control, indicated by higher HbA1c, is strongly associated with DH. These findings suggest that DH is a distinct complication of T1D, driven more by hyperglycemia than traditional vascular risk factors. Further research is needed to elucidate pathophysiological mechanisms and therapies.

**Supplementary Information:**

The online version contains supplementary material available at 10.1007/s42000-025-00732-5.

## Introduction

 Diabetes mellitus (DM) is a chronic metabolic disorder with an increasing global prevalence [1, 2]. While vascular complications are well-characterized, musculoskeletal manifestations are also common, yet often overlooked [3]. Notably, musculoskeletal hand pathology is a particularly overlooked complication, despite its clinical relevance due to its high prevalence, significant impact on quality of life, and substantial socio-occupational consequences [4, 5].

The term “diabetic hand” (DH) has lacked a consistent definition regarding the pathologies it includes and no clear consensus exists to date. Initially introduced in the late 20th century, the term predominantly referred to osteoarticular abnormalities associated with longstanding and poorly controlled diabetes, including Dupuytren’s contracture, trigger finger, and limited joint mobility (also known as diabetic cheiroarthropathy) [6, 7]. Later, carpal and cubital tunnel syndromes were added. Some authors, however, extend the term “diabetic hand” to include dermatological conditions, such as tropical diabetic hand syndrome, as well as infections, peripheral neuropathy of the upper limbs, and nail pathologies [4].

Although these conditions may occur in non-diabetic individuals, their incidence and prevalence significantly increase in the context of diabetes. Prevalence estimates vary depending on the study and the specific hand condition included. Reported ranges are approximately 8–50% for diabetic cheiroarthropathy, 20–63% for Dupuytren’s contracture, 11–16% for carpal tunnel syndrome, 10–15% for trigger finger [8], and 2–5% for cubital nerve compression [9]. While the underlying pathophysiological mechanisms remain incompletely elucidated, DH has been associated with factors such as microvascular complications, advanced age, and poor glycemic control, among other factors [3, 7, 10].

We conducted this study to analyze the characteristics associated with diabetic hand in a cohort of individuals with type 1 diabetes mellitus (T1D) using a case-control design to compare subjects with similar baseline variables.

## Materials and methods

This study is a retrospective case-control analysis conducted at a tertiary care hospital in Spain. Among a cohort of 968 individuals diagnosed with T1D aged over 18 years and actively followed up in the hospital’s dedicated diabetes outpatient services, 45 cases of DH were identified. Each case was matched with two controls based on age, sex, diabetes duration, and the presence of retinopathy, resulting in a final sample of 135 subjects.

### Inclusion criteria

For the purposes of our study, the definition of diabetic hand encompassed diagnoses made by specialists in endocrinology, rheumatology, or orthopedics, including at least one of the following conditions: carpal tunnel syndrome, flexor tenosynovitis (“trigger finger”), diabetic cheiroarthropathy, Dupuytren’s contracture, and De Quervain’s tendinitis. Diagnoses were primarily clinical and established using the following criteria:

- Dupuytren’s contracture: Presence of nodules or cords at the palmar or digital level, contractures, or skin retraction.
- Diabetic cheiroarthropathy (limited joint mobility): Presence of the “prayer sign” (inability to approximate the palmar surfaces of the proximal or metacarpophalangeal joints).
- Carpal tunnel syndrome: Pain, numbness, or paresthesia from the thumb to the radial side of the ring finger, with a positive provocative test (Tinel’s and/or Phalen’s signs) or confirmation via electromyography.
- Trigger finger: Presence of locking or clicking during finger flexion-extension, along with restricted phalangeal movement.
- De Quervain’s tendinitis: Pain over the radial styloid with a positive Finkelstein test.
- Cubital nerve compression: Pain, numbness, or paresthesia of the fourth and fifth digits and ulnar aspect of the hand, with a positive provocative test (Tinel’s sign) or confirmation via electromyography.

### Exclusion criteria

Individuals under 18 years of age, those with a prior diagnosis of rheumatoid arthritis, osteoarthritis of the hand, or traumatic hand conditions, and individuals with other forms of diabetes mellitus were excluded.

### Data collection

The data collected comprised diagnosis and year of diagnosis, coexisting hand pathologies, need for surgical intervention and its timing, and use of corticosteroid injections. Additionally, information on bilaterality and recurrence of hand pathology was recorded. Demographic variables included age, sex, body mass index (BMI, kg/m²), duration of diabetes, and cardiovascular risk factors (smoking, alcohol use, hypertension, dyslipidemia, and the intensity of lipid-lowering therapy per the 2019 ACC/AHA guidelines [11]). The type of diabetes treatment (multiple daily insulin injections or continuous subcutaneous insulin infusion) and the presence of microvascular (retinopathy, nephropathy, or distal symmetric polyneuropathy) and macrovascular complications (presence of ischemic heart disease, acute cerebrovascular events, acute pulmonary edema, peripheral artery disease, and diabetic foot) were also documented. Smoking was defined as consuming at least one cigarette, cigar, or pipe per day (excluding electronic cigarettes), and ex-smokers were defined as those who previously met this criterion but had quit by the time of the study.

Laboratory variables included the mean of the three most recent glycated hemoglobin (HbA1c) values before DH diagnosis (or, for controls, the values closest to the matched case’s diagnosis date), lipid profile (total cholesterol, LDL-C, HDL, and triglycerides), and renal profile (glomerular filtration rate estimated using CKD-EPI and microalbuminuria classified per KDIGO 2024 guidelines [12]). Diabetic retinopathy was categorized based on the International Clinical Diabetic Retinopathy Severity Scale [13] and recorded as present or absent. HbA1c was routinely measured using high-performance liquid chromatography (ADAMS A1c HA8180 V ARKRAY^®^), and cholesterol was analyzed using enzymatic methods (Alinity C Cholesterol Reagent Kit, Abbott).

Continuous glucose monitoring metrics near the diagnosis of DH were also included, such as average glucose (mg/dL), time in range (70–180 mg/dL), time below range (< 70 mg/dL), time above range (> 180 mg/dL), and glucose variability coefficient.

### Statistical analysis

Quantitative variables were expressed as mean and standard deviation if they followed a normal distribution and median and interquartile range if they did not. Normality of continuous variables was assessed using the Shapiro-Wilk test. As all variables followed a normal distribution, results are presented as mean ± standard deviation. Qualitative variables were presented as absolute frequencies and percentages.

To explore the relationship between HbA1c and the presence of diabetic hand, a LOESS curve (locally estimated scatterplot smoothing) was constructed to visually assess the association.

To address the lack of randomization and minimize confounding bias, a propensity score matching method was applied. Cases were matched to two controls using nearest-neighbor matching without replacement. Propensity scores were calculated using logistic regression models, including age, sex, diabetes duration, and retinopathy presence as covariates. This approach resulted in 45 cases matched to 90 controls, comprising a total sample of 135 individuals.

Bivariate differences between cases and controls were conducted using Student’s t-tests or Mann-Whitney U tests for quantitative variables and chi-square tests for categorical variables. For comparisons of normally distributed quantitative variables across multiple categories, ANOVA was used; for non-normal distributions, the Kruskal-Wallis test was applied. A conditional logistic regression model for matched data was developed, adjusting for covariates that were not well-balanced in univariate analyses and that could influence the development of DH.

Statistical analyses were performed using STATA version 17.0 BE-Basic Edition (Lakeway Drive, College Station, TX, USA). A p-value < 0.05 was considered significant.

### Ethics

The study protocol was approved by the local Research Ethics Committee and conducted in accordance with the Declaration of Helsinki relating to human studies (Study number 024–5691). The study adhered to the “Strengthening the Reporting of Observational Studies in Epidemiology (STROBE)” guidelines [14].

## Results

### Patient characteristics

**Fig. 1:**
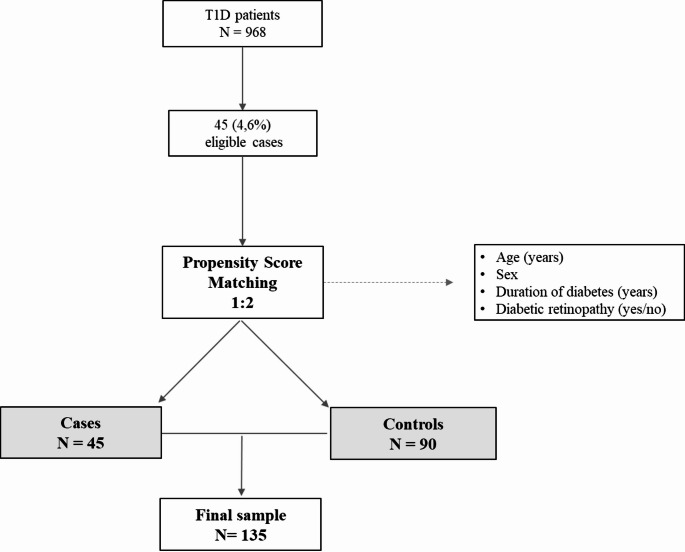
Flowchart. T1D: type 1 diabetes mellitus. Among the initial sample of individuals with T1D (968 participants), 45 individuals with hand pathology were identified. Each case was matched with two controls, resulting in a final sample of 135 subjects.

After applying the predefined inclusion and exclusion criteria, a total of 968 individuals with T1D under follow-up were selected. Among them, 45 individuals (4.6%) were identified with hand pathology. The flow diagram is shown in Fig. 1. Each case was matched with two controls based on age, sex, diabetes duration, and presence or absence of retinopathy. The final sample included 135 individuals, comprising 45 cases and 90 controls.

Demographic characteristics of the individuals are presented in Table 1. The mean age of the participants was 59.4 ± 16.5 years, and 74.8% were women. The mean diabetes duration was 31.7 ± 13.9 years, and 17% of the individuals were active smokers. The mean HbA1c was 7.75 ± 1.12%, with an average BMI of 25.3 ± 3.8 kg/m². Of the total sample, 46.7% exhibited some degree of diabetic retinopathy and 10.4% had nephropathy. Other complications included symmetric peripheral neuropathy in 20.8% of individuals and cardiovascular disease in 14.2%. Table 2 summarizes continuous glucose monitoring (CGM) data. The mean time in range (70–180 mg/dL) was 59.8 ± 19.4%, the mean time above range was 35.8 ± 20.3%, and the coefficient of variation was 36.1 ± 6.7. Table 3 outlines the characteristics of hand pathology. Among the 45 individuals with DH, 20 (44.4%) had at least two distinct hand pathologies. The most frequent diagnosis was carpal tunnel syndrome (68.8%), predominantly bilateral (70.9%), followed by trigger finger (51.1%). Only three individuals were diagnosed with cubital nerve compression and diabetic cheiroarthropathy and one patient with De Quervain’s tendinitis. Bilateral hand pathology was present in 60% of cases and approximately half of the cases (48.9%) required surgical intervention, with 13.6% needing reoperation. Additional variables are detailed in Tables 1, 2 and 3.

**Table 1:** General characteristics of the individuals included in the study. BMI: body mass index. DPN-L: diabetic lower limb polyneuropathy. HbA1c: glycated hemoglobin. (1) T-test, (2) Ji-squared test, (3) Wilcoxon test

	Obs *n* = 135	Control *N* = 90	Cases *N* = 45	*P* value
Age (years)	59.4 ± 16.5	58.9 ± 17.7	60.6 ± 13.8	0.571^1^
Sex (women)	101 (74.8%)	70 (77.8%)	31 (68.9%)	0.262^2^
Hypertension	59 (43.7%)	44 (48.8%)	15 (33.3%)	0.086^2^
Smoking habit	23 (17.0%)	14 (15.6%)	9 (20.0%)	0.517^2^
BMI (Kg/m²)	25.3 ± 3.8	24.9 ± 3.6	25.8 ± 4.06	0.220^1^
Duration of diabetes (years)	31.7 ± 13.9	31.1 ± 14.3	32.9 ± 13.3	0.481^1^
Diabetic retinopathy (%)	43 (46.7%)	44 (48.8%)	19 (42.2%)	0.464^2^
Diabetic nephropathy	14 (10.4%)	13 (14.4%)	1 (2.2%)	0.028^2^
DPN-L	14 (20.8%)	14 (15.6%)	6 (13.3%)	0.713^2^
Cardiovascular disease	19 (14.2%)	18 (20.2%)	1 (2.2%)	0.005^2^
Previous average HbA1c (%)	7.67 (6.93–8.3)	7.53 (6.8–8.13)	7.8 (7.2–8.67)	0.033^3^
Hypolipidemic treatment				
Non hypolipidemic	60 (44.4%)	34 (37.8%)	26 (57.8%)	0.027^2^
Low-potency	7 (5.2%)	3 (3.3%)	4 (8.9%)	0.170^2^
Moderate-potency	37 (27.4%)	26 (28.9%)	11 (24.4%)	0.585^2^
High-potency	47 (23.0%)	27 (30.0%)	4 (8.9%)	0.006^2^

**Table 2:** Characteristics of continuous glucose monitoring parameters 1. Wilcoxon test

	Obs *n* = 135	Control *N* = 90	Cases *N* = 45	*P* value
Time in rage (70–180 mg/dL)	62 (47–76)	62.5 (49–78)	60 (47–67)	0.1793^1^
Time above range (> 180 mg/dL)	33 (20–48)	32 (19–46)	34 (21–49)	0.339^1^
Time below range (< 70 mg)	3 (1–7)	3 (1–7)	3 (1–7)	0.729^1^
Coefficient of variation	36.1 ± 6.7	36.1 ± 7.0	35.9 ± 5.8	0.950

**Table 3:** Characteristics of the different hand pathologies. CT = carpian tunnel, cubital = cubital compression, TF = trigger finger, LJM = limited join mobility

	Obs *n* = 45	CT *N* = 31	TF *N* = 23	CubitalN=3	LJMN=3	De QuervainN=1
Surgery	22 (48.89%)	15 (48.4%)	15 (65.2%)	0	0	0
Bilateral	27 (60%)	22 (70.96%)	14 (60.87%)	1 (33.3%)	3 (100%)	0
Corticoid infiltration	10	4 (12.9%)	10 (43.48%)	0	2 (66.67%)	0
Surgical reintervention	3	1 (3.2%)	3 (13.04%)	0	0	0

### Hand pathology

A comparative analysis between the two groups revealed no significant differences in the matched variables: sex (68.9% women in the case group vs. 77.8% in controls), age (60.6 ± 13.8 years vs. 58.9 ± 17.7 years), presence of retinopathy (42.2% vs. 48.8%), and diabetes duration (32.9 ± 13.3 years vs. 31.1 ± 14.3 years). However, HbA1c was significantly higher in the case group (8.03 ± 1.3% vs. 7.6 ± 1.0%; *p* = 0.038).

A higher prevalence of cardiovascular disease was observed in the case group compared to controls (20.2% vs. 2.2%; *p* = 0.005), along with a greater proportion of individuals receiving high-intensity lipid-lowering therapy (30.0% vs. 8.9%; *p* = 0.006). Although there was a trend toward a lower time in range among the case group compared to controls (57.1 ± 17.7% vs. 61.2 ± 20.1%), this difference was not significant (*p* = 0.253). No significant differences were found for the remaining variables analyzed.

### Multivariable analysis

**Fig. 2:**
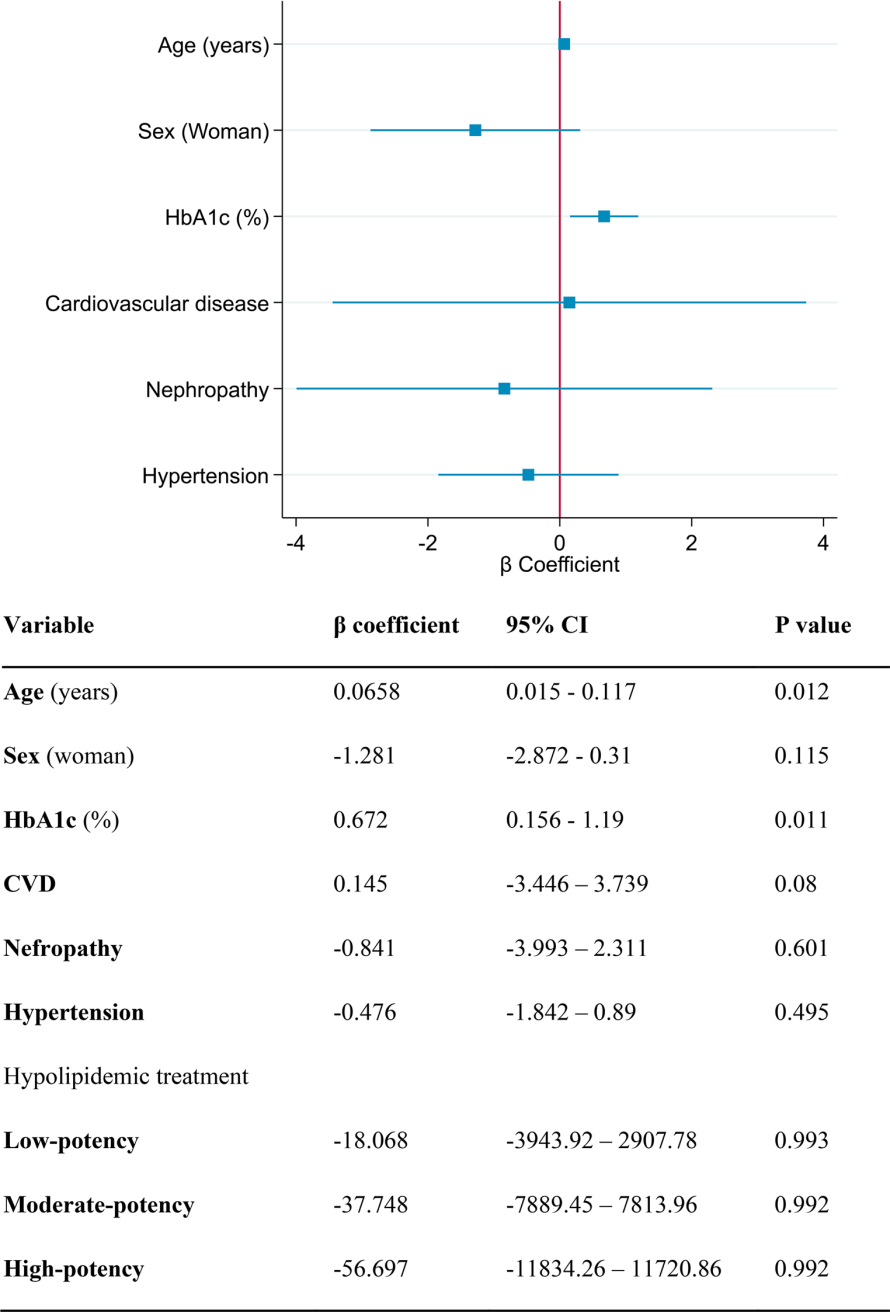
Association between hand pathology and clinical variables. HbA1c: glycated hemoglobin; CVD: cardiovascular disease. Multivariable analysis showing the association between hand pathology and selected clinical variables, including HbA1c, cardiovascular disease, and the intensity of lipid-lowering therapy. HbA1c and age were significantly associated with the presence of diabetic hand, while other variables such as nephropathy, hypertension, and CVD did not show significant associations after adjustment

A multivariable conditional logistic regression model for matched data was performed: the results are depicted in Fig. 2. The only variables significantly associated with the presence of diabetic hand were HbA1c (odds ratio [OR] 1.96, 95% confidence interval [CI] 1.16–3.28, *p* = 0.011) and age (OR 1.06, 95% CI 1.01–1.12, *p* = 0.012). We observed an approximately linear relationship between HbA1c and the presence of diabetic hand (Supplementary Figure S1). The model was adjusted for age, sex, and covariates showing differences between cases and controls, including nephropathy, hypertension, cardiovascular disease, and the intensity of lipid-lowering therapy. Interaction terms for cardiovascular disease and lipid-lowering therapy intensity were also included due to their strong interdependence, although neither was significant.

## Discussion

This study aimed to investigate factors associated with DH in a cohort of individuals with T1D by comparing cases and controls matched by baseline characteristics. Our findings demonstrate that glycemic control, as assessed by HbA1c, and age are the only variables significantly associated with DH. In contrast, cardiovascular risk factors did not influence its development. Although both cardiovascular disease and lipid-lowering therapy were more frequent among controls, neither showed an independent association after adjustment. These findings suggest that DH is not a vascular complication akin to other traditional diabetic manifestations, but rather one primarily associated with chronic poor glycemic control.

Hand pathologies in diabetes mellitus have attracted growing attention in recent years due to its prevalence and its detrimental impact on individuals’ quality of life. Since the initial reports of DH, the conditions encompassed by this term have varied, complicating study standardization. In our research, we incorporated the most prevalent pathologies described in prior studies, including carpal tunnel syndrome, diabetic cheiroarthropathy, trigger finger, Dupuytren’s contracture, cubital tunnel syndrome, and De Quervain’s tendinitis.

Recent studies suggest that etiopathogenic mechanisms contributing to DH, while variable, share a common underlying process. Chronic hyperglycemia triggers physiological pathways that can result in local fibrosis and potentially neuronal dysfunction, promoting neuropathy [15]. Advanced glycation end products (AGEs) have been proposed as being a key mechanism. AGEs may alter collagen by cross-linking it, causing structural and functional changes in periarticular connective tissues, leading to fibrosis and joint stiffness [16–18]. More recent research highlights the role of cellular senescence, suggesting that the diabetic cellular microenvironment promotes tissue-specific cellular senescence in mesenchymal stem cells and fibroblasts, contributing to local fibrosis [19–21]. Growth factors associated with fibrosis and cell proliferation, such as TGF-β and VEGF, along with proinflammatory metabolites in DH patient tissues, may perpetuate a state of inflammation and subsequently fibrosis, consistent with the aforementioned mechanisms [22, 23].

The relationship between DH and microvascular complications remains poorly understood. While several studies demonstrate associations between DH and various microvascular complications (e.g., retinopathy or nephropathy) [24–27], the evidence is not robust, as most studies are retrospective and involve small patient samples. Factors such as diabetes duration (regardless of type), age, and BMI have been consistently associated with DH [10, 28]. Both age and diabetes duration are closely related to DH development and have been repeatedly identified in prior research. From a pathophysiological perspective, these variables reflect prolonged exposure to a hyperglycemic tissue environment. For instance, studies on cheiroarthropathy indicate that it rarely manifests before 5–10 years after diabetes onset, underscoring the relationship between disease exposure and complication prevalence [29].

Data on glycemic control are less consistent. Recent studies, such as Yamamoto et al. in T1D populations [30] and Mustafa et al. in type 2 DM populations [31], suggest that DH is associated with diabetes duration and age rather than HbA1c, aligning with earlier studies [32]. However, as with most studies, heterogeneity in patient age and diabetes duration introduces potential confounding. In this context, matching cases and controls based on these variables is critical to minimizing bias from pre-existing differences.

In our study, supported by findings from other researchers [33–35], HbA1c and age were significantly associated with DH after matching. Although not significant, continuous glucose monitoring metrics showed trends consistent with HbA1c. In contrast, despite matching for retinopathy presence, no differences were observed between cases and controls, suggesting that DH may not behave as a microvascular complication, but rather has a distinct pathophysiology. This implies that glycemic control, as measured by HbA1c, independently and strongly associates with DH, highlighting its central role in the condition’s etiopathogenesis. This finding supports the hypothesis that chronic hyperglycemia contributes to DH development via mechanisms such as AGE accumulation and tissue damage induced by oxidative stress and chronic inflammation.

Management of hand pathologies should be individualized based on the specific condition. Approaches may include conservative measures such as rehabilitation and corticosteroid injections or surgical intervention when necessary. Early, multidisciplinary, and comprehensive management is essential to improve functional outcomes. In well-controlled diabetes, conservative measures are often effective, resolving the pathology without the need for surgery. For example, in Yamamoto et al.’s study [30], 26% of individuals achieved DH resolution within 1 year without orthopedic intervention. In our cohort, 34.8% of individuals with trigger finger and 51.1% with carpal tunnel syndrome did not require surgical treatment.

However, this study has several limitations. First, it is a single-center, retrospective study with an inherent sample selection bias. Second, the cohort design carries an inherent risk of observation and confounding biases. These potential biases were minimized by applying a propensity score matching protocol. Third, an important limitation of this study is the absence of certain potentially relevant variables that could influence the development of diabetic hand, such as occupational hand use, genetic susceptibility, and systemic inflammatory markers [36]. Fourth, the present study did not evaluate functional impairment or quality of life in individuals with DH. Finally, the diagnosis of neuropathy and other diabetes-related complications relied on the review of existing medical records rather than standardized or objective diagnostic testing.

## Conclusion

Chronic poor glycemic control appears to be independently associated with the presence of diabetic hand. Our study suggests that DH is not a manifestation linked to other microvascular complications. Further large-scale prospective studies are needed to identify additional specific risk factors and enhance the comprehensive management of these pathologies.

## Supplementary Information

Below is the link to the electronic supplementary material.

Supplementary Material 1 (DOCX 27.4 KB)
