# Supplementary material for: Hand pathology in type 1 diabetes mellitus: a case-control study
Source: Hormones (Athens). 2025 Nov 10;25(1):153–60. doi: 10.1007/s42000-025-00732-5 (PMC13013187; doi:10.1007/s42000-025-00732-5)
Supplement: Supplementary file 1 — Supplementary Material 1 (DOCX 27.4 KB) [file 42000_2025_732_MOESM1_ESM.docx]

**HORM-D-24-00457**

**SUPPLEMENTARY MATERIAL**

Supplementary Figure S1. LOESS curve depicting the relationship between HbA1c (%) and the probability of having diabetic hand. A nearly linear association is observed, suggesting that higher HbA1c levels are progressively associated with an increased risk of developing diabetic hand.
